# Comparative Effectiveness of Operative and Nonoperative Management Strategies for Acute Achilles Tendon Rupture: A Narrative Review

**DOI:** 10.7759/cureus.107485

**Published:** 2026-04-21

**Authors:** Adeolu A Badejo, Michael O Kolade, Timilehin Olowookere, Opeyemi Oyeleke, Kenechukwu Igbokwe, Imri G Adefokun, Funbi Ayeni

**Affiliations:** 1 Orthopaedic Surgery, Royal Victoria Infirmary, Newcastle upon Tyne, GBR; 2 General Surgery, Scarborough General Hospital, Scarborough, GBR; 3 Oral and Maxillofacial Surgery, South Eastern Health and Social Care Trust, Belfast, GBR; 4 Trauma and Orthopaedics, Stepping Hill Hospital, Stockport, GBR; 5 Trauma and Orthopaedics, Newcastle Upon Tyne NHS Foundation Trust, Newcastle upon Tyne, GBR; 6 Surgery, Bowen University, Iwo, NGA; 7 Orthopaedic Surgery, Bowen University Teaching Hospital, Ogbomoso, NGA; 8 Trauma and Orthopaedics, Imperial College Healthcare NHS Trust, London, GBR

**Keywords:** achilles repair, achilles rupture, achilles tendon injury, comparative effectiveness, functional rehab, nonsurgical treatment

## Abstract

Acute Achilles tendon ruptures (AATRs), an extremely prevalent injury amongst adults who remain active, have been treated with surgical intervention for decades. This choice was made due to several decades-old studies demonstrating reduced re-rupture rates as opposed to nonsurgical interventions utilizing casts; however, numerous surgeons and researchers acknowledged potential complications such as wound problems and nerve damage. Functional braces and early weight-bearing now challenge this 'surgical by default' model. An evidence-based, focused review of randomized clinical trials, conventional and network meta-analysis, and large cohorts was conducted for the comparison of operative and nonoperative treatment for AATRs or the evaluation of various rehabilitation approaches. Operative management provides a lower rate of re-rupture in comparison to traditional cast-based nonoperative treatment; however, operative management also results in increased wound complications and sural nerve injuries. Nonoperative management employing functional bracing and early weight-bearing provides re-rupture rates approaching those of operative management, and the long-term functional outcome data are generally comparable. Differences in structural changes continue to exist between the two management groups, with longer tendon length and atrophy of the soleus muscle seen after conservative treatment, although for the majority of patients, the resultant strength and endurance deficits will be minimal. Both treatment options may provide optimal results if implemented through structured rehabilitation pathways. Treatment should be individualized based on the patient's specific factors, injury characteristics, and available resources. The use of current comparative effectiveness research to assist in making decisions regarding treatment with the patient is recommended.

## Introduction and background

Acute Achilles tendon rupture (AATR) is primarily a clinical diagnosis, typically identified by sudden posterior ankle pain, weakness in plantarflexion, and a palpable tendon defect. A positive Thompson test, in which squeezing the calf fails to produce passive plantarflexion of the foot, is considered a key diagnostic finding.

AATRs are one of the most common and debilitating of all lower extremity injuries encountered by orthopedic surgeons as well as sports medicine physicians; an estimated population incidence of 18-24 per 100,000 person-years has been documented [1]. Physically active men aged 30-50 years are the predominant demographic group affected by this type of injury. Typically, they occur when patients experience a sudden eccentric load on their Achilles tendon while participating in recreational sports [2]. Incidence has dramatically increased over the past few decades as a result of a dramatic increase in middle-aged adults who are engaged in sports and/or leisure activities.

Optimal treatment of AATRs has been debated for many decades [3]. Traditional open surgical repair was preferred due to the lower re-rupture rate associated with it; however, the concern for postoperative wound infection, sural nerve injury, and anesthesia risk stimulated an interest in alternative methods of treatment (nonoperative) [4]. Traditional nonoperative management involved prolonged immobilization in equinus casting, which was associated with higher re-rupture rates and delayed functional recovery.

In recent years, treatment paradigms have evolved significantly. Modern nonoperative protocols now incorporate early functional rehabilitation, early weight-bearing, and controlled mobilization, which have demonstrated outcomes comparable to operative repair in several randomized controlled trials (RCTs) [5,6]. Techniques for minimally invasive treatment were also developed to reduce wound complications, resulting in a three-way equipoise that continues to exist today [4]. The purpose of this review is to synthesize 35 peer-reviewed studies (published from 2016 to 2026) that have examined the comparative effectiveness of operative and nonoperative management of AATRs, including functional outcome measures, re-rupture rates, complication rates, return to sports data, and optimizing rehabilitation.

Method

PubMed was searched in January 2026 using MeSH terms and keywords: "Achilles tendon rupture," "operative," "nonoperative," "functional rehabilitation," "re-rupture," and "return to sport," restricted to January 2016-March 2026. Peer-reviewed English-language articles were eligible; case reports, biomechanical studies, and nonhuman studies were excluded. A total of 35 articles, including RCTs, systematic reviews, meta-analyses, cohort studies, narrative reviews, and network meta-analyses, were selected for relevance and methodological quality. Because this study was conducted as a narrative review, a formal risk-of-bias tool was not applied. Instead, methodological quality was considered qualitatively by prioritizing higher levels of evidence, including RCTs, meta-analyses, and prospective comparative studies.

## Review

Epidemiology and mechanism of injury

Men experience an average of five times the number of Achilles tendon ruptures (ATRs) as women, with the majority of cases occurring between the ages of 40 and 49 years old [7]. Video analysis of professional basketball players revealed that the rupture occurred while pushing off in a noncontact manner when their foot was in a dorsiflexed position, and their knee was bent [2]. The data further indicate that nearly 37% of elite athletes who suffered an ATR did not return to competitive athletic play or retired from sports altogether because of the high degree of functional impairment [2]. The use of fluoroquinolones and the administration of systemic corticosteroids have been identified as potential risk factors for the degeneration and rupture of the Achilles tendon [8].

RCT evidence

Myhrvold et al. conducted the largest RCT to date on the treatment of ATRs, enrolling 554 patients in three treatment groups: nonsurgical treatment, open repair, and minimally invasive surgery [9]. All groups had significant improvements in function; however, there were no statistical differences among the groups in terms of the improvement in the Achilles tendon total rupture score (ATRS) at one year (p = 0.57). There were also significant differences in the rates of re-rupture, with the nonsurgically treated patients having a significantly higher re-rupture rate (6.2%) than either of the surgically treated groups (0.6%) [9]. There was also a significant difference in the rates of sural nerve injury, with the patients treated with minimally invasive surgery experiencing a significantly higher rate (5.2%) than those treated with either open repair (2.1%) or nonsurgical treatment (2.1%) [9].

Ochen et al. conducted a systematic review and meta-analysis of 29 RCTs involving 2060 patients treated with either operative or nonoperative treatment for an acute ATR [10]. The pooled results of this meta-analysis demonstrated a statistically significant difference in the rate of re-rupture (operative: 3.9%; nonoperative: 9.8%), but no difference in functional outcomes between the two treatment options [10]. Additionally, the meta-analysis demonstrated that operative treatment resulted in a statistically significant increase in complications compared to nonoperative treatment (operative: 15.8%; nonoperative: 5.0%) [10]. Furthermore, the authors of this meta-analysis found that early functional rehabilitation reduced the risk of re-rupture and narrowed the gap in complications between operative and nonoperative treatments.

Reito et al. conducted a meta-analysis of studies involving nonoperative patients receiving early functional rehabilitation [11]. This meta-analysis included a total of seven studies and found no statistically significant difference in the rate of re-rupture between operative and nonoperative treatment (operative: 3.0%; nonoperative: 4.8%) [11]. The results of this meta-analysis showed that operative treatment resulted in a statistically significant increase in complications compared with nonoperative treatment [11].

Fan et al., Yang et al., and Xu et al. conducted separate meta-analyses demonstrating that operative treatment significantly decreases the risk of re-rupture [12-14]. The results of these meta-analyses also indicate that standardized early rehabilitation achieves functional outcome equivalence to operative treatment.

Comparative effectiveness: observational and registry data

Crook et al. conducted a large propensity score-matched cohort study with 15,476 patients in each treatment group to compare operative and nonoperative treatments for acute ATR [15]. The results of this study demonstrate a significantly lower rate of re-rupture in the operative group (operative: 4.1%; nonoperative: 11.3%) [15]. Conversely, the results of this study also demonstrate a significantly higher rate of complications in the operative group (operative: 8.2%; nonoperative: 3.1%) [15].

Pisano et al. conducted a comprehensive network meta-analysis using data from 82 different treatment arms and 5,566 patients [16]. The results of this study identify open surgical repair combined with early weight-bearing as the optimal combination for reducing the risk of re-rupture while allowing for the fastest recovery [16]. Conversely, the results of this study identify nonsurgical treatment as the treatment modality with the greatest risk of re-rupture (12%) and deep vein thrombosis/pulmonary embolism [16].

Additionally, a previous study found that functional scores did not differ significantly between operative and nonoperative treatment groups at midterm follow-up [17]. However, the results of this study do indicate a significantly greater calf strength deficit in the nonoperative group (15% vs. 8%) [17]. Operative repair has been shown to result in superior tendon morphology (i.e., shorter and less elongated tendons); however, this superior tendon morphology does not result in measurable differences in functional performance between the two treatment modalities at 12+ months post-injury [18].

Operative techniques comparison

Melinte et al. conducted a systematic review and meta-analysis comparing mini-open and percutaneous repair of an acute ATR [19]. The results of this meta-analysis demonstrated that the mini-open repair method resulted in a significantly lower rate of re-rupture (mini-open: 1.48%; percutaneous: 6.11%; odds ratio (OR): 0.28; p = 0.03) and a significantly lower rate of sural nerve injury (mini-open: 0.57%; percutaneous: 5.64%; OR: 0.24; p = 0.02) [19]. Furthermore, a comparison of percutaneous knotless and open repair of an acute ATR at a mean five-year follow-up demonstrated no significant differences in Functional Activities Measure (FAM) Ankle Hindfoot Scale-Activities of Daily Living (FAAM ADL), FAM Ankle Hindfoot Scale-Sports, or patient satisfaction [20]. However, the results of this study demonstrated that the percutaneous repair method required no revisions, whereas the open repair method required two revisions [20].

Additionally, a study comparing two minimally invasive techniques, Percutaneous Achilles Repair System (PARS) and Midsubstance Speedbridge (MSB), in 119 patients demonstrated equivalent scores in Patient-Reported Outcome Measurement Information System (PROMIS) Physical Function (PARS: 58.8 vs MSB: 55.3) and ATRS (PARS: 86.0 vs MSB: 82.5) at 30 months, indicating equivalence of the two minimally invasive methods [21]. Lastly, a prospective study of percutaneous repair reported a 25% complication rate, predominantly transient sural nerve injuries, and supports conservative management as a first-line option for cooperative younger patients [22].

Return to sport and patient-reported outcomes

A systematic review by Cofano et al. showed that 77.4% of surgically treated patients were able to return to sport at a mean of 8.1 months after surgery. Their postoperative AOFAS and Tegner scores also exceeded those expected for the general population, as did their low re-rupture rate of 2.3% [23]. A systematic review by Bak et al. on return to play found that operative treatments allowed quicker return to sport than nonoperative treatments (mean: 6.5 vs 8.2 months; standardized mean difference: -0.42; 95% confidence interval (CI): -0.71 to -0.13). However, they also noted no difference in long-term functionality [24]. Using PROMIS, Burger et al. also showed slightly better physical function scores at 12 months in the operative group (53.2 vs 49.8; p = .03). They also noted a significantly lower re-rupture rate in the operative group (1.5% vs 9.1%; p = .04) [25].

Rehabilitation protocol and weight-bearing strategies

Meta-analyses have consistently shown that early weight-bearing during nonoperative treatment does not increase the risk of re-rupture [26]. Ghaddaf et al. reviewed 10 RCTs (n = 1112) and found that early weight-bearing resulted in a significant decrease in time to return to work (mean difference: -2.6 weeks; p < 0.01) and time to return to sport (mean difference: -3.1 weeks; p < 0.01) with no increase in re-rupture risk (OR: 0.90; 95% CI: 0.52-1.57) [27]. Nonoperative rehabilitation programs using accelerated rehabilitation techniques such as equinus positioning, dynamic functional splinting, and structured physiotherapy achieved re-rupture rates of 3-6% and allowed return to sport at approximately 20 weeks [28]. An immediate full weight-bearing program following surgical repair of the tendon resulted in a significant improvement in ATRS score (from 29.5 to 79.3 points; p < 0.01) and a low major complication rate of 3.5% [29].

Complications and venous thromboembolism (VTE) risk

The UK FATE prospective multicentre study (n = 1247 patients who underwent Achilles repair) reported a 30-day VTE rate of 2.7%. The primary risk factors identified included BMI > 30 (OR: 2.4), immobilization for greater than six weeks (OR: 3.1), and previous VTE history (OR: 4.2) [30]. The authors found that chemical thromboprophylaxis resulted in a 49% relative risk reduction in VTE (OR: 0.51; 95% CI: 0.28-0.93), which supports its targeted use in high-risk patients regardless of whether or not they are undergoing operative treatment [30].

Discussion

Studies have consistently shown that both the operative and nonoperative management of acute ATRs can produce similar functional results as long as early functional rehabilitation is implemented consistently [9,11,31]. Much of the recent paradigm shift towards earlier postoperative rehabilitation has been due to the recognition that it is the postoperative rehabilitation process, not the surgical procedure itself, that drives most functional recovery [5,6]. There is considerable evidence that the historical association of reduced re-rupture rates following surgical repair is greatly diminished, and in some cases, no longer present, when early postoperative weight-bearing functional bracing programs are used to manage nonoperatively [11,27].

Although there is a demonstrated similarity in functional outcomes, the differences in complication rates between the two treatment groups remain a concern to clinicians. Postoperative complications associated with operative repair of an acute ATR include wound infection, sural nerve damage, deep vein thrombosis, and the potential risks associated with anesthesia. Nonoperative management of an acute ATR has been associated with a re-rupture rate of approximately 6-12% in the pooled literature, particularly if the rehabilitation program is suboptimal [10,12,32]. Therefore, based on the current literature, it is imperative to select patients appropriately for either operative or nonoperative management of an acute ATR.

Younger, highly active athletic individuals, those who present with greater than a 10 mm gap between the ends of the ruptured tendon on ultrasonography, or those who are at low surgical risk, may choose operative repair of their acute ATR for the potential re-rupture benefit and quicker return to competitive sports [33,34]. There is also no evidence in the literature supporting the superiority of one operative technique over another. Studies have shown that open repair, mini-open repair, and percutaneous repair yield similar functional scores, although percutaneous repair is associated with a significantly higher incidence of sural nerve injury [4,19]. Mini-open repair appears to provide the optimal balance between reduced re-rupture rates and sural nerve injury rates compared to other operative techniques, while minimizing the size of the wound relative to traditional open approaches [19]. Additionally, emerging literature comparing the use of different minimally invasive implant systems (i.e., PARS versus MSB) for repair of acute ATRs indicates that these systems produce similar clinical outcomes [21].

Acute myotendinous junction ruptures are a distinct subgroup of acute ATRs. While there is limited evidence suggesting that operative repair of acute myotendinous junction ruptures may result in fewer re-rupture events than nonoperative repair, the clinical relevance of this difference remains uncertain [35]. Regardless of the chosen treatment pathway, all patients with acute ATRs should receive chemoprophylaxis against VTE. A prospective study reported a 30-day VTE rate of 2.7%, which was decreased by 49% using targeted chemical prophylaxis (Table 1) [30].

**Table 1:** Comparative effectiveness of operative and nonoperative management of acute Achilles tendon rupture; summary of 35 peer-reviewed articles ATR: Achilles tendon rupture; MIS: minimally invasive surgery; WB: weight-bearing; EWB: early WB; LWB; late WB; ATRS: Achilles tendon total rupture score; AOFAS: American Orthopaedic Foot & Ankle Society score; PROMIS: Patient-Reported Outcomes Measurement Information System; FAAM: Foot and Ankle Ability Measure; RTP/RTS: return to play/sports; ROM: range of motion; DVT: deep vein thrombosis; PE: pulmonary embolism; OR: odds ratio; RCT: randomized controlled trial; N/A: not applicable

#	First author(s)	Year	Study type/design	Treatment comparison	N	Key findings	Clinical implication
1	Myhrvold et al. [9]	2022	Multicentre RCT	Nonoperative vs open repair vs MIS	526	No difference in ATRS at 12 months (p = 0.57). Re-rupture: nonoperative 6.2% vs both surgical groups 0.6%. Nerve injury is highest in the MIS group (5.2%)	Surgery is not superior for function; it reduces re-rupture at the cost of nerve injury risk
2	Ochen et al. [10]	2019	Systematic review & meta-analysis (29 RCTs)	Operative vs nonoperative	2,060	Pooled re-rupture: 3.9% vs 9.8%. Complications: 15.8% (op) vs 5.0% (nonop). No difference in functional scores. Early rehab reduced re-rupture in both groups	Early functional rehab narrows re-rupture gap; complication trade-off must guide decisions
3	Attia et al. [4]	2022	Systematic review & meta-analysis of RCTs	Open repair vs minimally invasive surgery	552	No differences in functional outcomes or re-rupture rates. MIS is associated with higher sural nerve injury (OR: 3.82; 95% CI: 1.11-13.14). Open repair had higher infection rates	Choose a technique based on nerve vs infection risk tolerance; functional outcomes are equivalent
4	Fan et al. [12]	2024	Meta-analysis of RCTs (14 studies)	Surgical vs nonoperative	1,854	Surgery significantly reduced re-rupture (OR: 0.24; 95% CI: 0.12-0.48). Functional scores and return-to-activity are similar. Overall complications were higher in the surgical group	Surgery prevents re-rupture; nonoperative is viable with proper rehabilitation
5	Pisano et al. [16]	2025	Systematic review & network meta-analysis	Open repair, MIS, nonoperative ± EWB/LWB	5,566	Open + LWB has the lowest re-rupture (2%). Nonsurgical: highest re-rupture (12%) and DVT/PE risk. Percutaneous repair increases sural nerve injury vs open (4% vs 1%)	Open repair is the gold standard for re-rupture prevention; EWB aids recovery
6	Xu et al. [14]	2025	Systematic review & meta-analysis (18 RCTs)	Operative vs nonoperative	2,156	Surgery reduced re-rupture (RR: 0.28; 95% CI: 0.17-0.46). Complications higher operatively (RR 2.95). ATRS and AOFAS equivalent at 1 year. Early rehab narrows re-rupture gap	With standardized early rehab, nonoperative management achieves clinical equivalence for most patients
7	Bak et al. [24]	2024	Systematic review & meta-analysis	Operative vs nonoperative (return-to-play focus)	3,476	RTP rate: 80% (op) vs 73% (nonop). Operative: faster RTP (6.5 vs 8.2 months; SMD: −0.42). ATRS equivalent at final follow-up	Surgery enables a faster return to play; long-term functional outcomes are equivalent
8	Crook et al. [15]	2023	Propensity score-matched cohort study	Operative vs nonoperative	30,952	Operative: lower re-rupture (4.1% vs 11.3%; OR: 0.34; p < 0.001) but higher complications (8.2% vs 3.1%). Time to return and satisfaction are similar between groups	Operative reduces re-rupture; complication risk must be weighed using shared decision-making
9	Reito et al. [11]	2022	Systematic review & meta-analysis (5 RCTs)	Operative vs nonoperative with early functional rehab	1,023	Re-rupture: 3.0% vs 4.8% (OR: 0.62; p = 0.32; not significant). Complications were significantly higher in the operative group (15.4% vs 5.4%; OR: 3.16)	When early rehab is universal, surgery offers no significant re-rupture advantage
10	Ge et al. [17]	2023	Systematic review (midterm follow-up)	Operative vs nonoperative	2,148	At 2-5 years: ATRS, AOFAS, and Tegner scores are not significantly different. Calf strength deficit was greater after nonoperative management (15% vs 8%). Re-rupture lower operatively (3.2% vs 8.9%)	Both approaches yield satisfactory long-term outcomes; residual calf strength deficit persists nonoperatively
11	Zhu et al. [18]	2024	Systematic review (22 studies)	Operative vs nonoperative (morphology & function)	22 studies	Operative repair produced shorter, less elongated tendons with a smaller cross-sectional area. Functional performance tests (hop tests, heel rise) did not differ significantly at 12+ months	Superior tendon morphology after surgery does not translate to better functional performance
12	Yang et al. [13]	2025	Systematic review & meta-analysis (20 RCTs)	Surgical vs conservative	2,412	Surgery: lower re-rupture (RR: 0.31; 95% CI: 0.19-0.50; p < 0.001). Complications more common surgically (RR: 2.71). No difference in ATRS, AOFAS, or return to activity	Management should be individualized; the re-rupture advantage of surgery diminishes with early rehab
13	Burger et al. [25]	2025	Prospective cohort study	Operative vs nonoperative (PROMIS outcomes)	123	Operative group: higher PROMIS Physical Function at 12 months (53.2 vs 49.8; p = 0.03). Re-rupture: 1.5% vs 9.1% (p = 0.04). PROMIS Pain Interference similar between groups	Operative provides modest functional and re-rupture advantage at 1 year by PROMIS metrics
14	Dai et al. [26]	2021	Systematic review & meta-analysis (8 RCTs)	Rehabilitation regimens in nonoperative treatment	978	No significant differences in re-rupture, return to sport/work, functional outcome, QoL, or complications between early weight-bearing with functional motion vs traditional immobilization	Early weight-bearing in a functional brace is a safe, cost-effective alternative to a non-weight-bearing cast
15	Ghaddaf et al. [27]	2022	Systematic review & meta-analysis (10 RCTs)	Early vs late weight-bearing in conservative management	1,112	Early WB did not increase re-rupture (OR: 0.90; 95% CI: 0.52-1.57). Faster return to work (−2.6 wk; p < 0.01) and return to sports (−3.1 wk; p < 0.01). DVT incidence similar	Early weight-bearing is recommended for all nonoperative patients-faster recovery, no added re-rupture risk
16	El-Akkawi et al. [6]	2018	Meta-analysis (5 RCTs)	Early vs late weight-bearing in conservative management	Not reported	No significant difference in re-rupture (p = 0.796), return to sports (p = 0.455), or return to work (p = 0.888). Early WB group showed improved QoL and dorsiflexion ROM	Early WB is safe and provides QoL and range-of-motion benefits without increasing re-rupture risk
17	Coopmans et al. [28]	2022	Systematic review (12 studies)	Accelerated nonoperative rehabilitation	1,086	Accelerated rehab: re-rupture rates 3-6%, return to work ~10.5 weeks, return to sports ~20.2 weeks. ATRS/FAOS showed marked improvement at 12 months	Accelerated nonoperative rehabilitation is safe and effective, with outcomes approaching those of surgical management
18	Won Lee et al. [29]	2022	Retrospective cohort study	Immediate full WB & ankle motion exercise after surgery	67	ATRS improved from 29.5 to 79.3 (p < 0.01). Return to sports: mean 20.6 weeks. Full squat in 92.8% at ~10 weeks. Major complication rate: 3.5% (1 re-rupture, 1 tendon elongation)	Immediate weight-bearing and early ankle motion after repair yield low complication rates and good outcomes
19	Bullock & Pierson [8]	2024	Narrative review	Nonoperative vs open repair vs MIS (comprehensive review)	N/A	Modern evidence supports equivalent functional outcomes with all three approaches when early functional rehab is applied. MIS reduces wound complications while maintaining functional equivalence	Functional rehabilitation protocol choice is as important as the surgical vs nonsurgical decision
20	Amendola et al. [1]	2022	Evidence-based narrative review	Diagnosis through nonoperative, open, and MIS treatment	N/A	Thompson test sensitivity: 96%; specificity: 93%. Ultrasound preferred for imaging. Both operative and nonoperative approaches achieve comparable outcomes with early rehab. MIS reduces wound complications	No single superior management strategy; evidence-based pathway from diagnosis to treatment presented
21	Glazebrook & Rubinger [5]	2019	Narrative review	Functional rehabilitation for nonoperative treatment	N/A	Early WB rehab: re-rupture 2-6%, calf strength recovery 85-95% at 12 months, return to sports 6-9 months. Structured patient-selection algorithm presented.	Accelerated functional rehab with early WB is the preferred nonoperative strategy
22	Mansfield et al. [7]	2022	Narrative review (athletes)	Nonoperative vs operative for athletes; return to play	N/A	Mean RTP: 6-8 months (operative) vs 8-10 months (nonoperative). Re-rupture: 2-4% (op) vs 6-12% (nonop). RTS rate >80% in elite athletes with either approach	Shared decision-making incorporating athlete-specific factors should guide treatment in sports medicine
23	King & Vartivarian [34]	2023	Narrative review	Primary repair to complex augmentation for ATR	N/A	Primary end-to-end repair standard for acute ATR. FHL transfer or allograft for chronic/degenerative ruptures. Percutaneous/mini-open preferred for reduced wound complications	Surgical complexity should be tailored to acuity, gap size, and tissue quality of the rupture
24	Aurich et al. [33]	2024	Semi-quantitative literature analysis	Operative vs nonoperative (10 meta-analyses, 2 cost analyses)	N/A	OP and N-OP lead to equally good function with early rehab. Lower re-rupture rate favours surgery; lower complications favour N-OP. M-OP shows an increased sural nerve lesion rate vs O-OP	M-OP with sural nerve protection combined with early mobilization is the authors' preferred approach
25	Cofano et al. [23]	2025	Systematic review (9 studies)	Surgical repair outcomes (return to sport focus)	748	RTS rate 77.4%; mean RTS time 8.1 months. Re-rupture rate 2.3%. Postoperative infection 3.3%. AOFAS and Tegner scores exceeded normative values at a mean 3.5-year follow-up	Surgical repair achieves high RTS rates with an acceptable complication profile
26	Melinte et al. [19]	2024	Systematic review & meta-analysis (8 studies)	Mini-open vs percutaneous surgical repair	484	Mini-open: lower re-rupture (1.48% vs 6.11%; OR: 0.28; p = 0.03) and sural nerve injury (0.57% vs 5.64%; OR: 0.24; p = 0.02). AOFAS was higher in mini-open group (MD +1.52; p = 0.001)	Mini-open technique preferred over percutaneous repair for a superior safety profile
27	Deng et al. [3]	2017	Systematic review & meta-analysis (11 RCTs)	Surgical vs conservative treatment	1,380	Surgery: lower re-rupture (OR: 0.29; 95% CI: 0.18-0.48; p <0.0001). Complications higher surgically (OR: 2.67). No functional outcome difference at 12 months. Early rehab attenuated the surgical re-rupture advantage	Treatment should be individualized; early functional rehabilitation narrows the gap between approaches
28	Meulenkamp et al. [31]	2018	Systematic review protocol (network meta-analysis)	All operative and nonoperative interventions	N/A	Protocol for network meta-analysis to simultaneously compare all ATR management strategies. Primary outcomes: re-rupture, overall complications, functional scores. Plans to rank interventions	Formal comparative ranking of all ATR strategies requires network analysis; protocol established
29	Cohen et al. [32]	2023	Evidence-based review / editorial	Operative vs nonoperative (evidence-based framework)	N/A	Multiple high-quality RCTs support equivalent functional outcomes. Lower complication rate favours nonoperative. Key surgical factors: young athletes, significant tendon gap, and patient preference	An evidence-based framework supports shared decision-making between the surgeon and the patient
30	Stake et al. [20]	2023	Retrospective cohort study	Percutaneous knotless repair vs open repair	61	No significant differences in FAAM ADL (99 vs 99), FAAM Sports, SF-12, or Tegner at the mean five-year follow-up. Operative time slightly longer percutaneously (52 vs 46 min). No revisions in the percutaneous group	Percutaneous knotless repair is a safe, effective, long-term alternative to open repair
31	Peabody et al. [21]	2025	Retrospective comparative study	PARS vs Midsubstance Speedbridge (MSB) MIS repair	119	No significant differences in PROMIS PF (58.8 vs 55.3), PROMIS PI (44.2 vs 44.0), or ATRS (86.0 vs 82.5) between PARS and MSB at a mean 30-month follow-up. Complication rates similar	PARS and MSB are equivalent MIS options; both achieve above-average PROMIS Physical Function scores.
32	Lameire et al. [35]	2025	Systematic review (8 studies)	Operative vs nonoperative for myotendinous ATR	219	53% managed operatively. Operative: lower re-rupture (0% vs 5.1%), higher functional scores. Complications more common operatively (8.2% vs 2.0%). Return to sports similar (~8-10 months)	Operative management may offer re-rupture advantage for myotendinous ATR; evidence is limited by small studies
33	Mangwani et al. [30]	2024	Prospective multi-centre observational study	VTE incidence and thromboprophylaxis after Achilles repair	1,247	30-day VTE rate: 2.7% (DVT: 1.9%; PE: 0.8%). Risk factors: BMI > 30 (OR: 2.4), immobilization >6 wk (OR: 3.1), prior VTE (OR: 4.2). Chemical prophylaxis reduced VTE by 49% (OR 0.51; p = 0.03)	Targeted chemical thromboprophylaxis is warranted in higher-risk patients undergoing Achilles tendon surgery
34	Lemme et al. [2]	2019	Descriptive epidemiology study	ATR epidemiology, mechanism, and outcomes in NBA athletes	44 ruptures	All ruptures were noncontact. Mean RTP: 10.5 months. 36.8% did not return to full play or retired. Player efficiency rating declined by 2.9 points (p < 0.001)	Elite athletes face significant career risk from ATR; early-season veteran players are the highest-risk group
35	Campillo-Recio et al. [22]	2023	Prospective cohort study	Percutaneous repair with absorbable suture	52	ATRS: 92.45 at 6 months, 94.04 at 12 months. Re-rupture: 5.77%. Total complication rate 25% (predominantly transient sural nerve injuries)	High complication rate (sural nerve) supports conservative treatment as first-line in suitable younger patients

Some of the limitations of the studies reviewed include variability in rehabilitation protocols, varying lengths of follow-up, mostly male study participants, and the lack of a comprehensive cost-effectiveness analysis of treatments. Future studies should prioritize standardized evaluation of patient outcomes, extended follow-up (beyond two years) of patients after treatment for acute ATRs, and the identification of patient-specific predictors of successful treatment.

## Conclusions

Operative and nonoperative management of AATRs result in equivalent functional outcomes when early functional rehabilitation programs are uniformly employed. While operative repair of AATRs decreases the frequency of re-rupture events, operative repair increases the number of postoperative complications and the potential for adverse effects of surgery. Thus, the decision regarding whether to use operative or nonoperative management for AATRs should be made individually, taking into account the patient's age, level of physical activity, medical comorbid conditions, likelihood of re-rupture events, ability to participate in supervised postoperative rehabilitation, and available resources. If a decision is made to use operative repair, then mini-open repair is preferred because of its favourable complication profile. Regardless of the treatment pathway chosen, VTE prophylaxis should be considered in patients identified as being at increased risk for developing VTE.
